# Long-term exercise results in morphological and biomechanical changes in coronary resistance arterioles in male and female rats

**DOI:** 10.1186/s13293-020-0284-0

**Published:** 2020-02-12

**Authors:** Marianna Török, Anna Monori-Kiss, Éva Pál, Eszter Horváth, Attila Jósvai, Petra Merkely, Bálint András Barta, Csaba Mátyás, Attila Oláh, Tamás Radovits, Béla Merkely, Nándor Ács, György László Nádasy, Szabolcs Várbíró

**Affiliations:** 1 Department of Obstetrics and Gynecology, Semmelweis University, Üllői u. 78/a, Budapest, 1082 Hungary; 2 Institute of Clinical Experimental Research, Semmelweis University, Tűzoltó u. 37-47, Budapest, 1094 Hungary; 3 Department of Neurosurgery, Military Hospital, Róbert Károly körút 44, Budapest, 1134 Hungary; 4 Heart and Vascular Center, Semmelweis University, Városmajor u 68, Budapest, 1122 Hungary; 5 Department of Physiology, Semmelweis University, Tűzoltó u 37-47, Budapest, 1094 Hungary

**Keywords:** Ventricular hypertrophy, Exercise, Resistance coronary arteries, Gender, Sex differences

## Abstract

**Background:**

Biomechanical remodeling of coronary resistance arteries in physiological left ventricular hypertrophy has not yet been analyzed, and the possible sex differences are unknown.

**Methods:**

Wistar rats were divided into four groups: male and female sedentary controls (MSe and FSe) and male and female animals undergoing a 12-week intensive swim training program (MEx and FEx). On the last day, the in vitro contractility, endothelium-dependent dilatation, and biomechanical properties of the intramural coronary resistance arteries were investigated by pressure microarteriography. Elastica and collagen remodeling were studied in histological sections.

**Results:**

A similar outer radius and reduced inner radius resulted in an elevated wall to lumen ratio in the MEx and FEx animals compared to that in the sedentary controls. The wall elastic moduli increased in the MEx and FEx rats. Spontaneous and TxA_2_ agonist-induced tone was increased in the FEx animals, whereas endothelium-dependent relaxation became more effective in MEx rats. Arteries of FEx rats had stronger contraction, while arteries of MEx animals had improved dilation.

**Conclusions:**

According to our results, the coronary arterioles adapted to an elevated load during long-term exercise, and this adaptation depended on sex. It is important to emphasize that in addition to differences, we also found many similarities between the sexes in the adaptive response to exercise. The observed sport adaptation in the coronary resistance arteries of rats may contribute to a better understanding of the physiological and pathological function of these arteries in active and retired athletes of different sexes.

## Background

Long-term regular exercise induces hypertrophy and remodeling of the left ventricular myocardium (‘athlete’s heart’). Cardiac hypertrophy following intensive sport is a physiological condition. The increased myocardial mass is associated with increased stroke volume and a lower resting heart rate [1, 2], without cardiomyocyte apoptosis, cardiac fibrosis, or changes in fetal gene expression [3–6]. This complex adaptive cardiac remodeling is called athlete’s heart. Exercise-induced cardiac hypertrophy is reversible, and cessation of training results in progressive morphological and functional regression [7, 8]. The elevated myocardial mass requires increased perfusion, which can be provided by remodeling of only the coronary circulation [5]. In contrast, pathological cardiac hypertrophy is induced by pressure or volume overload (e.g., hypertension or valvular disorder) [9]. Pathological cardiac hypertrophy is associated with apoptosis and necrosis of cardiomyocytes, enhanced interstitial fibrosis, and reactivation of the fetal cardiomyocyte gene program [4, 5]. In pathological conditions, the initial phase of remodeling is a compensatory response to the increased biomechanical stress that acts to maintain normal cardiovascular function [10]. This condition decompensates and leads to ventricular dilatation, which can further lead to systolic and diastolic myocardial dysfunction (heart failure) [3].

The size of the coronary arteries can be expected to be an important factor in the sex differences induced by exercise training, as there are substantial structural and functional differences between the different artery types in the heart. The conducting and distributing arteries have large diameters (> 400 μm) and are flexible due to the presence of elastin in the artery wall. Their primary function is to transfer blood to smaller resistance coronary vessels. In contrast, resistance vessels have small diameters (< 200 μm), and their walls are composed mainly of smooth muscle, so they are able to contract actively in response to metabolic, hormonal and neuronal stimuli. Their primary function is to regulate local hemodynamic resistance and to ensure the oxygen requirements of different heart areas. Most studies that have described the remodeling of the coronary arteries in exercise-induced ventricular hypertrophy, however, deal with large subepicardiac vessels, while the number of publications on resistance arteries is limited because of methodical difficulties [11–13].

A paper on the effects of a *moderate* chronic treadmill exercise program on intramural coronary arterioles in *male* rats was recently published. In the low intraluminal pressure range, the distensibility and endothelium-dependent modulation of myogenic tone were augmented, whereas at higher pressures, wall thickness increased, wall stress was reduced, the myogenic response increased, and the effect of intrinsic constrictor prostanoids was diminished [14].

Differences between male and female vascular function and disease risk are now well established. Some sex-specific characteristics of resistance artery function were previously published by our group [15, 16]. Sex differences regarding several aspects of cardiovascular adaptation in response to physical exercise have previously been proven [17, 18]. There is good reason to think that such differences exist in the coronary resistance artery system as well. The present study investigated whether the resistance coronary arteries are adapted structurally and functionally to long-term intensive exercise during the process in which the ventricular myocardium is transformed into ‘athlete’s heart.’ Furthermore, our intention was to determine whether there are sex differences in this adaptation process of intramural coronary resistance arteries to long-term, intensive physical exercise and what type of differences can be observed. Presently, no study is available on coronary resistance artery remodeling induced by long-term intense physical exercise comparing both sexes.

## Materials and methods

### Animals

Young adult (*n* = 32, 12 weeks old) male and female Wistar rats were housed in a room with constant temperature (22 ± 2 °C) with a 12-h light-dark cycle. They were kept on a standard laboratory rat diet provided ad libitum and had free access to water.

Throughout the experiments, all animals received care in compliance with the Principles of Laboratory Animal Care formulated by the National Society for Medical Research and the ‘Guide for the Care and Use of Laboratory Animals’ prepared by the Institute for Laboratory Animal Resources and published by the National Institutes of Health (NIH Publication No. 86-23, revised 1996). All procedures and handling of the animals during the study were approved by the Animal Care Committee of Semmelweis University as well as by state authorities (permission number: PEI/001/2374–4/2015).

### Chemicals

Pentobarbital (Euthasol, CEVA Santé Animale, Liboume, France) was used for anesthesia (45 mg/kg i.p.). The composition of the normal Krebs-Ringer (Krebs buffer) solution used in these in vitro studies was as follows (mM): 119 NaCl, 4.7 KCl, 1.2 NaH_2_PO_4_, 1.17 MgSO_4_, 24 NaHCO_3_, 2.5 CaCl_2_, 5.5 glucose, and 0.0345 EDTA. The calcium-free Krebs solution (buffer without Ca^2+^) contained 92 NaCl, 4.7 KCl, 1.18 NaH_2_PO_4_, 20 MgCl_2_, 1.17 MgSO_4_, 24 NaHCO_3_, 5.5 glucose, 2.0 EGTA, and 0.025 EDTA. The temperature of the solution was kept at 37 °C, and it was bubbled with 5% CO_2_, 20% O_2_, and 75% N_2_ that stabilized the pH at 7.4. Salts were obtained from Reanal (Budapest, Hungary). U46619, L-NAME, and bradykinin acetate (with the purity of all chemicals being greater than 98%) were obtained from Sigma-Aldrich (St. Louis, Missouri, US).

Frozen aliquots were diluted each day.

### Experimental groups and the intensive swim training protocol

After 1 week of acclimatization, the animals were randomly divided into four groups: male exercised (MEx, *n* = 8), female exercised (FEx, *n* = 8), male sedentary control (MSe, *n* = 8), and female sedentary control (FSe, *n* = 8). The groups in training (MEx and FEx) underwent a graded, intense swimming exercise protocol [1]. Water is a physiological medium for rats, and the swimming abilities of these rodents are excellent. The animals were placed in a container of water (separately; the container was divided into six lanes with a depth of 45 cm and a surface area of 20 × 25 cm per lane) with smooth walls that were filled with moderately warm water (30–32 °C). The rats swam in their own lanes, and the dimensions of the lanes were selected to prevent any reclining against the walls. The program started with 15 min of swimming per day, and the time of exercise was raised every second day by an additional 15 min until the duration of swimming reached a total of 200 min, which was then maintained throughout the experiment. Trained rats swam for a total of 12 weeks with 5 days of swimming + 2 days of rest per week. The control sedentary groups (MSe and FSe) were only put in the water for 5 min daily, 5 days/week, in parallel with the 12-week-long training program of the swimmers. The body weight and general shape of the animals were regularly monitored. There were no animals lost or any complications encountered during the training program, and all the animals were healthy throughout the experimental period.

### Echocardiography

Echocardiographic assessments were performed after completion of the training program, as described previously [2]. The transthoracic echocardiography investigation was performed under isoflurane anesthesia (1–2% isoflurane in 100% oxygen) using a 13 MHz linear transducer (GE, Healthcare, Horten, Norway) attached to a Vividi Echocardiac Image Analysis System (GE, Healthcare, US). Standard two-dimensional short-axis records were acquired (at the mid-papillary level). The stored pictures were analyzed by blinded investigators using EchoPac v113 (GE, Healthcare software). The left ventricular end-diastolic and end-systolic diameters (LVEDD and LVESD, respectively) and the anterior and posterior wall thicknesses (AWT and PWT, respectively) in diastole were measured at the mid-papillary level on two-dimensional short-axis pictures. The computed parameters were fractional shortening [(FS) = (LVEDD–LVESD)/LVEDD*100], and ejection fraction (EF), computed according to the Teichholz method, (EF) = (LVEDV-LVESV)/LVEDV*100 [19].

### In vitro pressure arteriography of intramural coronary arteries

At the end of week 12, under pentobarbital anesthesia (45 mg/kg body weight, intraperitoneal), blood pressure was measured via cannulation of the right carotid artery (Gould pressure heads), and the animals were perfused with 150 ml of saline to remove all blood from the vessels. The chest was opened, the heart was removed, and the weight of the heart was measured. Thereafter, in a cold Krebs-Ringer solution, from an intramural branch of the left anterior descending coronary artery, resistance-sized arteries with an outer diameter of 200 μm [16] were prepared by careful microdissection under a preparation microscope (Wild, M3Z, Leica, Olympus Heerbrugg, Switzerland), as described previously [20]. The arteriolar segment with a length of approximately 2 mm was placed in a tissue bath with a glass bottom filled with a normal Krebs-Ringer solution (37 °C). The arteriolar segment was cannulated at both ends with plastic microcannulas of 130 μm. The original in situ length of the segment was fixed by axial screws. The segments were pressurized using servo-controlled roller pumps (Living Systems, Burlington, VT, US). Continuous superfusion with a volume of 2.8 ml/minute was ensured, while the total volume of the tissue bath was 12 ml. The bath was placed on the stage of an inverted microscope (Leica), and magnified pictures of the mounted, pressurized segments were taken with a DCM 130 E camera. Pictures were taken regularly and stored. The analysis of the pictures was performed with specific image-analyzing software (ScopePhoto). The inner and outer diameters and wall thicknesses were measured. A length calibration was performed with a micrometer etalon (Wild, Heerbrugg, Switzerland).

To study the biomechanical properties of coronary resistance arteries, the following protocol was used. Arteries from sedentary and swim-trained male and female rats were taken and incubated in Krebs buffer solution at 50 mmHg of intraluminal pressure for 30 min. Resistance-sized arteries develop spontaneous contraction if they are incubated in an oxygenated medium [21]. To induce the initial contraction, no contracting agent was added. A pressure diameter curve was then determined by increasing the pressure from 0 to 150 mmHg in 50 mmHg steps. The steady-state diameter was measured at each step. Thereafter, bradykinin (BK) was added in cumulative concentrations (10^−8^, 10^−7^, and 10^−6^ M, each concentration lasted for 10 min), and the diameters were measured. Then, the NO synthase blocker nitro-l-arginine methyl ester hydrochloride (L-NAME) was added (10^−5^ M) for 20 min, and the diameters were measured again. Drugs were washed out, and after a 10-min rest, the original diameter was restored, and we added U46619, a TxA_2_ receptor agonist, (at a concentration of 10^−7^ M) to the bath; the vessel was then incubated for 10 min, and the pressure diameter curves were recorded repeatedly. To test reproducibility, U46619 was washed with Krebs buffer, followed by incubation for 20 min in Krebs buffer. Vessels with myogenic tone differing from the original by more than 5% at this point were rejected. Finally, the segments were incubated for 30 min in a buffer without Ca^2+^, and the inner and outer diameters were measured in the passive state at 50 mmHg. Then, the pressure diameter curves were recorded repeatedly to compute the incremental elastic moduli and tangential wall stress.

The biomechanical parameters were calculated as follows: the wall/lumen ratio, *Q* = *h*/*d*_i_; the wall stress, σ = (P*r_i_)/*h*), according to the Laplace-Frank equation; the wall thickness, *h* = *r*_o_-*r*_i_; and the incremental tangential elastic modulus of the cylindrical segments, *E*_inc_ = (2*r*_o_*r*_i_^2^*Δ*P*)/((*r*_o_^2^–*r*_i_^2^)*Δ*r*_o_), where *r*_o_ and *r*_i_ are the actual values of the outer and inner radii, *d*_i_ is the inner diameter, *P* is the transmural (intraluminal) pressure, and Δ*r*_o_ is the alteration of the outer radius during a pressure rise of Δ*P*, according to Cox [22].

From the pressure-diameter data, the following parameters were calculated:

- Spontaneous tone: *T*_Krebs buffer_ = 100 × (*r*_obuffer withoutCa2+_ − *r*_oKrebs buffer_)/*r*_obuffer withoutCa2+_(%);
- Bradykinin-induced relaxation: *T*_BK_ = 100 × (*r*_oBK_ − *r*_oKrebs buffer_) / *r*_oKrebs buffer_*100(%)
- L-NAME-induced contraction: *T*_L-NAME_ = 100 × (*r*_oBK_ − *r*_oL-NAME_) / *r*_oKrebs buffer_ (%)
- U46619-induced contraction: *T*_TxA2_ = 100 × (*r*_obuffer withoutCa2+_ − *r*_oTxA2_) / *r*_oKrebs buffer_ (%),

where *r*_oKrebs buffer_ and *r*_obuffer withoutCa2+_ are the outer radii measured in a normal Krebs-Ringer solution and a calcium-free solution at the same pressure. *r*_oBK_, *r*_oL-NAME,_ and *r*_oTxA2_ are the outer radii measured after bradykinin, L-NAME, and the TxA_2_ agonist (U46619) at the same pressure, respectively.

### Histology studies

The segments used for the biomechanical measurements and the whole heart were removed and placed in 4% formaldehyde for fixation (*n* = 4–4). After dehydration, they were embedded in paraffin, and 5-μm-thick sections were cut. The elastin fibers and noncontractile fibers were stained with resorcin-fuchsin (RF) on the coronary segments. The collagen networks were stained with picrosirius (PS), and the amount of smooth muscle was stained with smooth muscle actin (SMA) on the heart sections. The sections were photographed with an unaltered setting of a Zeiss Axiometer digital microscope using a × 20 objective (pixel sizes 0.27 μm). The resorcin-fuchsin-stained sections were analyzed by colorimetric techniques to evaluate the inner elastic membrane, as described previously [23]. RGB pictures at the 0–255 level were analyzed with Leica QWin software. The green levels (suppressed by the magenta color of the resorcin dye) were checked in radial lines from the endothelial surface towards the adventitia. The noncalibrated optical density of specific staining (RF, PS, and SMA) was measured by ImageJ software (NIH, Bethesda, MA, USA). In the case of heart sections, only coronary arteries 100–400 μm in diameter were evaluated.

### Statistical evaluation

SPSS Sigma Stat software was used. Data are presented as the mean ± SEM. Normal distribution was tested with the Shapiro-Wilks method. A two-way analysis of variance (ANOVA) with the factors ‘training’ and ‘sex’ was performed. If there were interactions between ‘training’ and ‘sex’ (*p*_int_ < 0.05) in the two-way ANOVA (body weight, heart weight, heart weight/body weight, L-NAME-induced contraction, optical density on RS staining), we used one-way ANOVA. As a post hoc test, Tukey’s post hoc test was used in both one-way and two-way ANOVA. A *P* value of < 0.05 was used as the criterion for statistical significance. GraphPad Prism 5 software was used to create the figures.

## Results

### Physiological alterations

When comparing the exercised male rats with untrained control male rats, MEx animals had less body weight gain during the 12-week-long study period, whereas weight gain did not differ between the female sedentary and female exercised groups (Table 1). Female rats (FSe and FEx) weighed less than the male rats (MSe and MEx) at both the start and at the end of the training program (Table 1). The postmortem measured heart weights were higher in FEx animals than in FSe rats; furthermore, the heart weights were higher in both male groups than in the corresponding females (Table 1). Postmortem measured heart weight values in proportion to body weight were higher in both exercised groups (Table 1). The heart weight/body weight ratio was significantly higher in FEx rats than in MEx rats (Table 1).

**Table 1:** Basic characteristic parameters of the study groups

Variable	Male sedentary	Male exercised	Female sedentary	Female exercised
Basic characteristics				
BW^1^ (g)	335 ± 9	331 ± 5	211 ± 2*	216 ± 3^$^
BW^2^ (g)	512 ± 15	427 ± 6*	287 ± 5*	272 ± 3^$^
HW (g)	1.71 ± 0.04	1.75 ± 0.07	1.03 ± 0.04*	1.31 ± 0.03^#$^
HW/BW^2^ (g/kg)	3.35 ± 0.08	3.92 ± 0.05*	3.59 ± 0.13	4.84 ± 0.14^#$^

BW^1^, body weight at the start of the training program (*F*_training_ = 0.0266, *F*_sex_ = 496.645, *F*_int_ = 0.783, df_training_ = 1, df_sex_ = 1, df_int_ = 1, *P*_training_ = 0.872, *P*_sex_ < 0.001, and *P*_int_ = 0.384); BW^2^, body weight at the end of the training program (*F*_between_ groups = 180.443, df_between_ groups = 3, P_between groups_ < 0.001); *HW* heart weight (*F*_between_ groups = 45.132, df_between_ groups = 3, *P*_between groups_ < 0.001); HW/BW^2^, heart weight/body weight ratio (*F*_between_ groups = 37.254, df_between_ groups = 3, *P*_between groups_ < 0.001)

One-way ANOVA (BW^2^, HW, HW/BW^2^) and two-way (BW^1^) ANOVA with post hoc Tukey’s test. Values are the means ± SEM. *P < 0.05 vs. male sedentary; ^#^*P* < 0.05 vs. female sedentary; ^$^*P* < 0.05 vs. male exercised

**Fig. 1:**
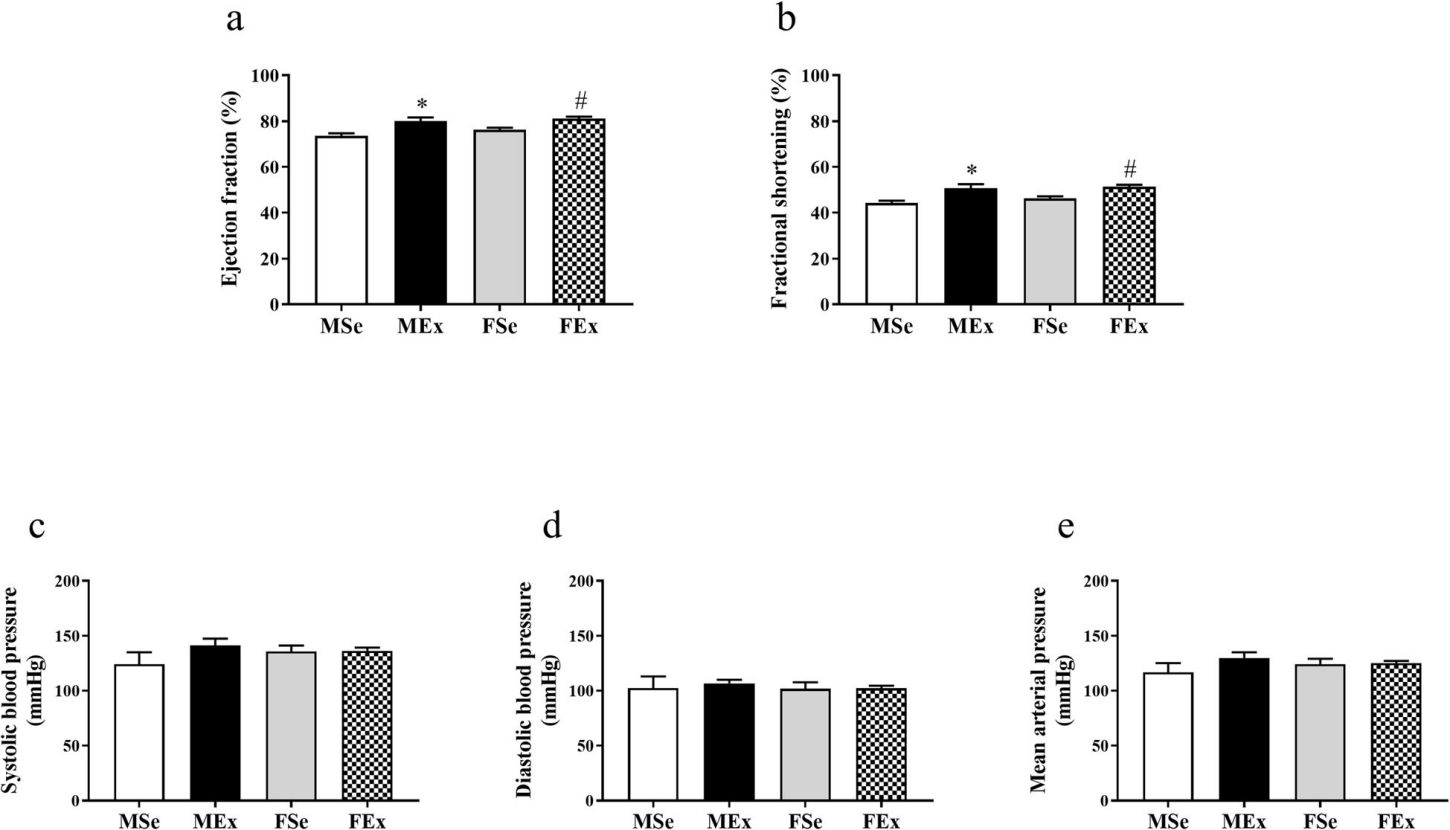
Exercise-induced alterations in cardiac function and blood pressure. **a** Long-term training significantly elevated the ejection fraction of the left ventricle in the MEx and FEx groups compared with the corresponding control groups, and no difference between the sexes was found. (*F*_training_ = 25.226, *F*_sex_ = 2.735, *F*_int_ = 0.495, df_training_ = 1, df_sex_ = 1, df_int_ = 1, *P*_training_ < 0.001, *P*_sex_ = 0.106, and *P*_int_ = 0.486). **b** Similar observations were made for fractional shortening. (*F*_training_ = 24.528, *F*_sex_ = 1.263, *F*_int_ = 0.402, df_training_ = 1, df_sex_ = 1, df_int_ = 1, *P*_training_ < 0.001, *P*_sex_ = 0.268, and *P*_int_ = 0.53). **c** No alterations were found in systolic blood pressure. (*F*_training_ = 1.593, *F*_sex_ = 0.219, *F*_int_ = 1.403, df_training_ = 1, df_sex_ = 1, df_int_ = 1, *P*_training_ = 0.218, *P*_sex_ = 0.644, and *P*_int_ = 0.246). **d** No alterations were found in diastolic blood pressure (*F*_training_ = 0.149, *F*_sex_ = 0.164, *F*_int_ = 0.0876, df_training_ = 1, df_sex_ = 1, df_int_ = 1, *P*_training_ = 0.703, *P*_sex_ = 0.688, and *P*_int_ = 0.769). **e** No alterations were found in mean arterial pressure (*F*_training_ = 1.473, *F*_sex_ = 0.0570, *F*_int_ = 1.241, df_training_ = 1, df_sex_ = 1, df_int_ = 1, *P*_training_ = 0.235, *P*_sex_ = 0.813, and *P*_int_ = 0.275). Two-way ANOVA with post hoc Tukey’s test. Values are the means ± SEM. *P < 0.05 vs. MSe; ^#^*P* < 0.05 vs. FSe

The systolic function of the enlarged ventricle (determined by the ejection fraction and fractional shortening) was significantly elevated in both males and females by long-term intensive swim training (Fig. 1a, b). There was, however, no significant difference in the systolic, diastolic, and mean arterial pressures (Fig. 1c–e).

### Histology studies

#### Resorcin-fuchsin staining

**Fig. 2:**
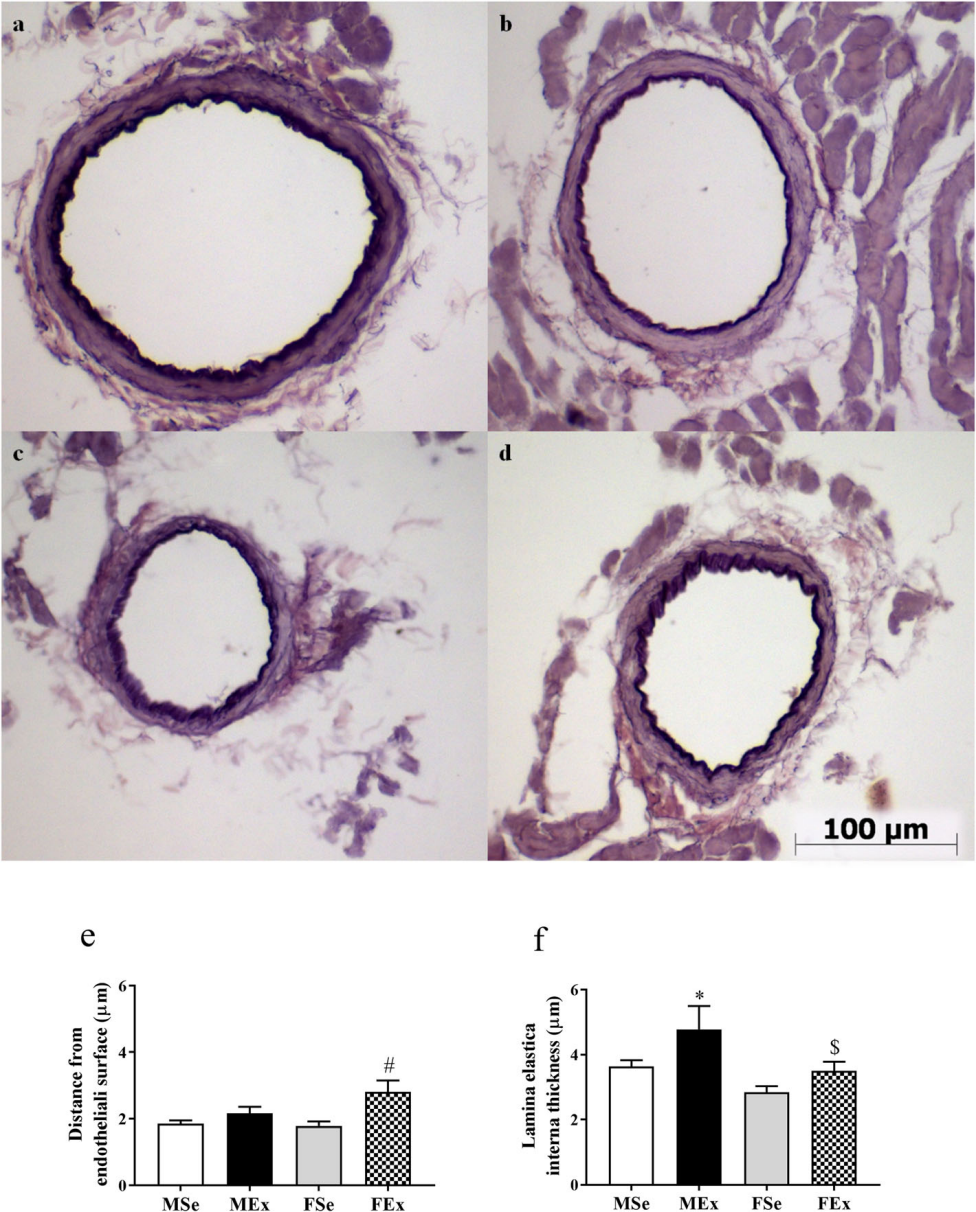
Elastica staining. **a** Representative staining in the MSe rats**. b** Representative staining in Mex rats**. c** Representative staining in FSe rats**. d** Representative staining in FEx rats**. e** The distance of the maximum density layer of the inner elastic lamina from the endothelial surface. It was significantly increased in the FEx group compared to the FSe group. (*F*_training_ = 9.47, *F*_sex_ = 1.795, *F*_int_ = 2.677, df_training_ = 1, df_sex_ = 1, df_int_ = 1, *P*_training_ = 0.01, *P*_sex_ = 0.104, and *P*_int_ = 0.128). **f** The thickness of the inner elastic lamina increased in the MEx animals in comparison with the male sedentary animals and with the trained female group. (F_training_ = 10.574, *F*_sex_ = 13.637, *F*_int_ = 1.355, df_training_ = 1, df_sex_ = 1, df_int_ = 1, *P*_training_ = 0.007, *P*_sex_ = 0.003, and *P*_int_ = 0.267). Two-way ANOVA with post hoc Tukey’s test. Values are the means ± SEM. *P < 0.05 vs. MSe; ^#^*P* < 0.05 vs. FSe; ^$^*P* < 0.05 vs. MEx

The characterization of the elastica staining (Fig. 2a–d) by the reduction in the green RGB component (suppressed by the magenta color of the resorcin dye) resulted in two observations. The distance of the layer with maximal intensity was located farther away from the endothelial surface in the trained female animals compared to that in the control female animals (Fig. 2e). The inner elastic membrane became thicker in the MEx animals than that in the MSe and FEx animals. There seemed to be a tendency toward thickening of the inner elastic lamina in the trained female animals, but the difference did not reach the level of statistical significance (Fig. 2f).

**Fig. 3:**
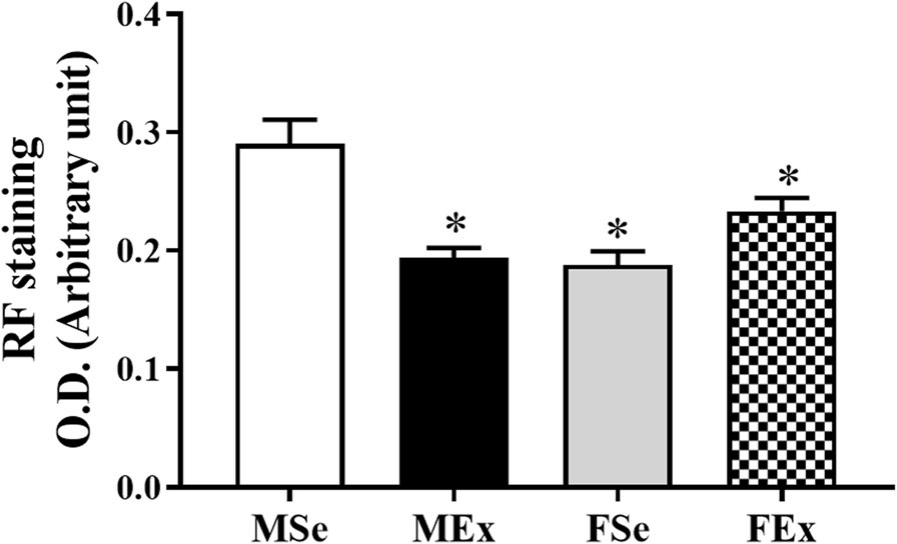
Optical density on resorcin-fuchsin-stained segments. The optical density was significantly lower in sedentary and trained female animals than in control male rats. The OD was significantly reduced in trained male animals compared to sedentary male animals. (*F*_between_ groups = 11.911, df_between_ groups = 3, *P*_between groups_ = < 0.001). One-way ANOVA with post hoc Tukey’s test. Values are the means ± SEM. *P < 0.05 vs. MSE

The optical density of noncontractile fibers on resorcin-fuchsin-stained sections was significantly lower in female animals (FSe and FEx) than in MSe rats, and this value was significantly reduced in the MEx group approaching the level of that in the female animals (Fig. 3).

#### Picrosirius and smooth muscle actin staining

**Fig. 4:**
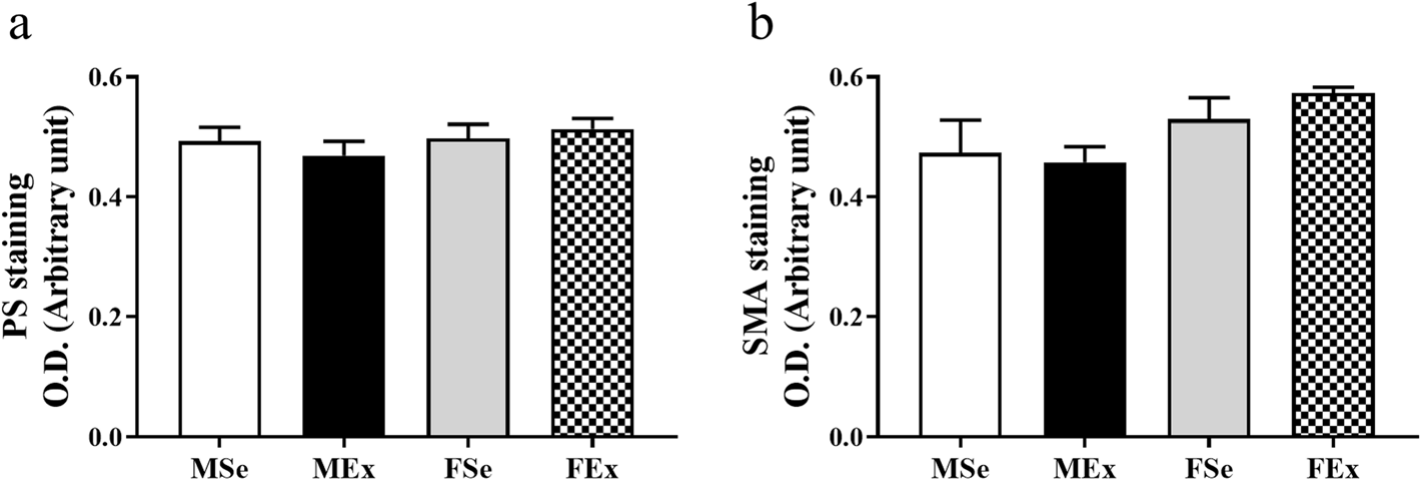
Picrosirius and smooth muscle actin staining of coronary resistance artery segments. **a** No alterations were found in PS staining. (*F*_training_ = 0.0332, *F*_sex_ = 1.186, *F*_int_ = 0.804, df_training_ = 1, df_sex_ = 1, df_int_ = 1, *P*_training_ = 0.859, *P*_sex_ = 0.299, and *P*_int_ = 0.389). **b** No alterations were found in SMS staining. (*F*_training_ = 0.136, *P*_sex_ = 4.892, *P*_int_ = 0.588, df_training_ = 1, df_sex_ = 1, df_int_ = 1, *P*_training_ = 0.719, *P*_sex_ = 0.047, and *P*_int_ = 0.458). Two-way ANOVA with post hoc Tukey’s test. Values are the means ± SEM

The optical density of collagen (PS staining) and smooth muscle actin (SMA staining) did not differ between the groups (Fig. 4a, b).

#### Contractility parameters for the intramural coronary resistance arteries from the physiologically hypertrophic left ventricle

**Fig. 5:**
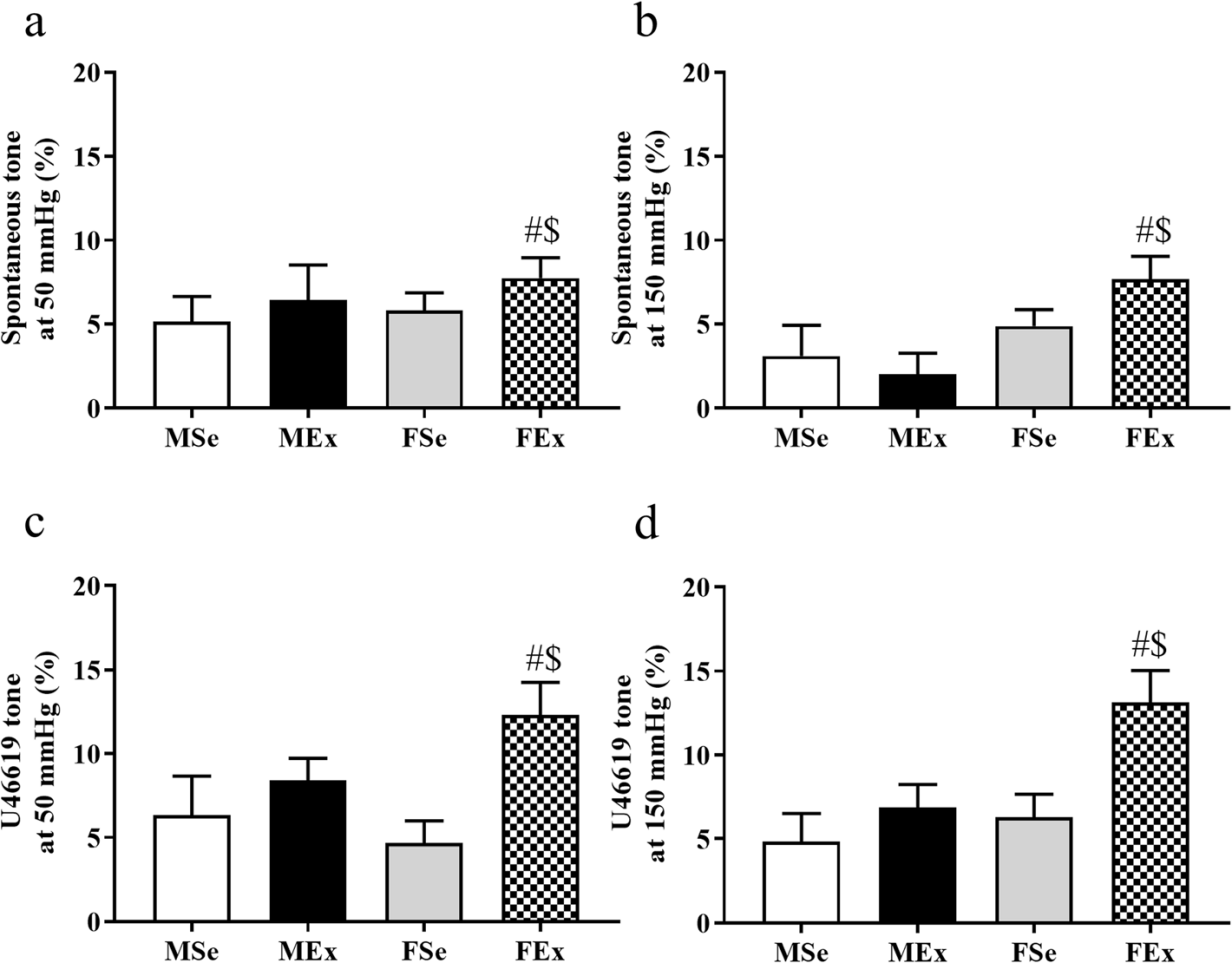
Contractility parameters of coronary resistance artery segments induced by long-term physical exercise. **a** No alterations were found at 50 mmHg for spontaneous tone. (*F*_training_ = 1.15, *F*_sex_ = 0.404, *F*_int_ = 0.0452, df_training_ = 1, df_sex_ = 1, df_int_ = 1, *P*_training_ = 0.293, *P*_sex_ = 0.53, and *P*_int_ = 0.833). **b** Spontaneous tone was significantly elevated in FEx rats compared with MEx rats at 150 mmHg. (*F*_training_ = 0.396, *F*_sex_ = 7.089, *F*_int_ = 1.9, df_training_ = 1, df_sex_ = 1, df_int_ = 1, *P*_training_ = 0.534, *P*_sex_ = 0.013, and *P*_int_ = 0.179). **c** The maximum contraction ability was tested with the TxA_2_ agonist U46619 (10^−7^ M). Training significantly increased contractility in the FEx group at 50 mmHg compared with the FSe group. (*F*_training_ = 7.481, *F*_sex_ = 0.405, *F*_int_ = 2.51, df_training_ = 1, df_sex_ = 1, df_int_ = 1, *P*_training_ = 0.011, *P*_sex_ = 0.53, and *P*_int_ = 0.124). **d** At 150 mmHg, U46619 contraction was significantly higher in the FEx animals than that in the FSe and MEx animals. (*F*_training_ = 7.668, *F*_sex_ = 5.827, *F*_int_ = 2.28, df_training_ = 1, df_sex_ = 1, df_int_ = 1, *P*_training_ = 0.01, *P*_sex_ = 0.023, and *P*_int_ = 0.142). Two-way ANOVA with post hoc Tukey’s test. Values are the means ± SEM. ^#^*P* < 0.05 vs. FSe; ^$^*P* < 0.05 vs. MEx

Spontaneous tone, measured in vitro at 50 mmHg, was not significantly affected by the 12-week-long swim training (Fig. 5a). However, at 150 mmHg, it was significantly higher in FEx than in MEx animals (Fig. 5b).

**Fig. 6:**
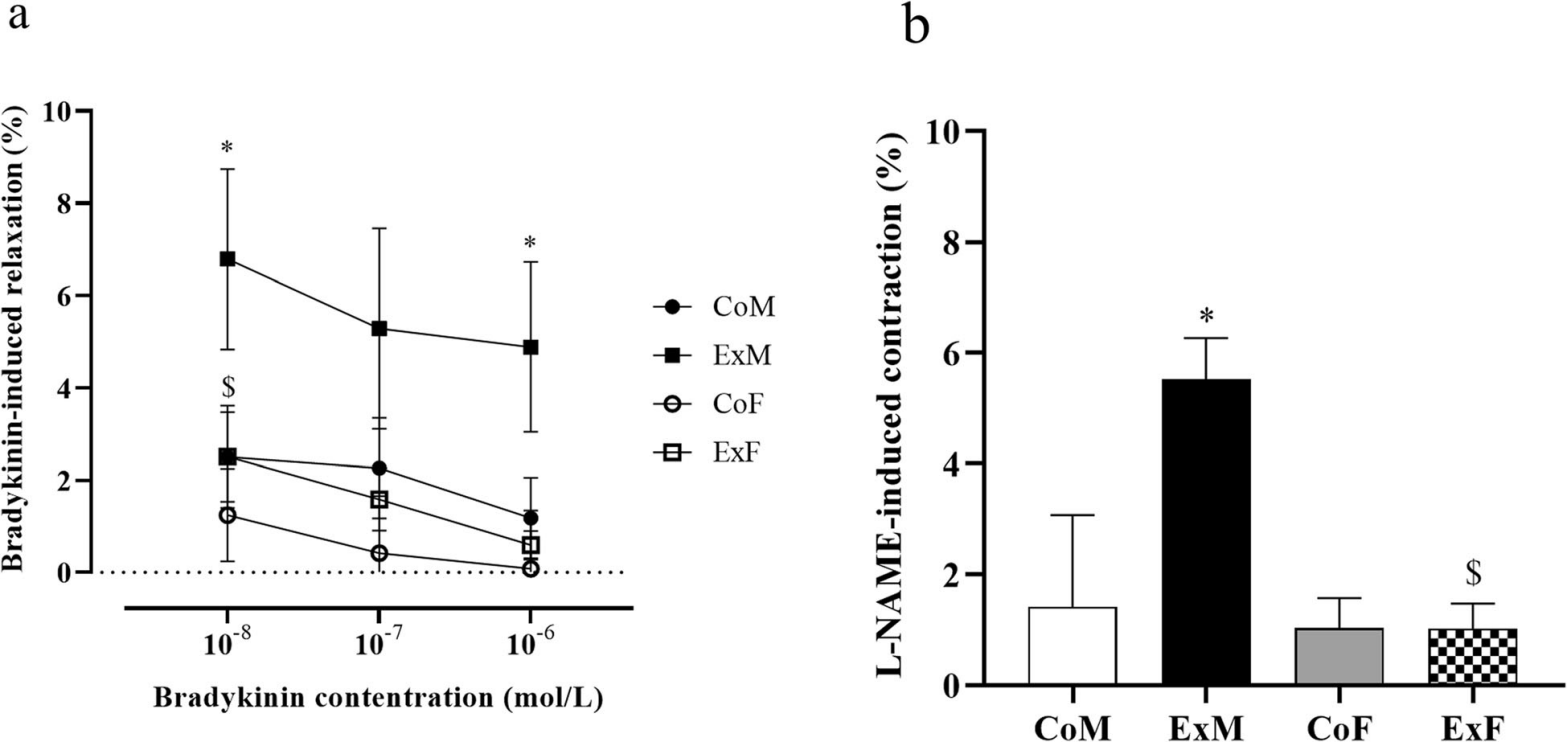
Contractile and relaxation properties of coronary resistance arteries induced by long-term physical exercise. **a** Relaxation induced by increasing concentrations of bradykinin at 50 mmHg pressure. (at 10^−8^ M: *F*_training_ = 4.391, *F*_sex_ = 4.374, *F*_int_ = 1.304, df_training_ = 1, df_sex_ = 1, df_int_ = 1, *P*_training_ = 0.045, *P*_sex_ = 0.046, and *P*_int_ = 0.263; at 10^−7^ M: *F*_training_ = 2.225, *F*_sex_ = 3.909, *F*_int_ = 0.44, df_training_ = 1, df_sex_ = 1, df_int_ = 1, *P*_training_ = 0.147, *P*_sex_ = 0.058, and *P*_int_ = 0.513; at 10^−6^ M: *F*_training_ = 3.047, *F*_sex_ = 4.99, *F*_int_ = 1.746, df_training_ = 1, df_sex_ = 1, df_int_ = 1, *P*_training_ = 0.092, *P*_sex_ = 0.034, and *P*_int_ = 0.197). **b** Contraction induced by L-NAME at 50 mmHg. (*F*_between_ groups = 5.091, df_between_ groups = 3, *P*_between groups_ = 0.006). One-way (L-NAME) and two-way (bradykinin) ANOVA with post hoc Tukey’s test. Values are the means ± SEM. *P < 0.05 vs. MSE, ^$^*P* < 0.05 vs. MEx

Training resulted in significantly better bradykinin-induced relaxation at 10^−8^ M and 10^−6^ concentrations in exercised male rats compared with control males (at 10^−7^ M, the relaxation did not reach a significant level (*p* = 0.058)). Relaxation induced by increasing concentrations of bradykinin was unaltered in control and trained female rats (Fig. 6a). In the presence of 10^−6^ M bradykinin, the NO production blocker L-NAME (10^−5^ M) induced significantly higher contraction in MEx animals than in MSe rats. Comparing the L-NAME-induced contraction in the FSe and FEx groups, we found equal values (Fig. 6b). Bradykinin-induced relaxation was greater in trained males than in trained female rats at 10^−8^ M (Fig. 5a). The contraction caused by L-NAME was also higher in MEx rats than in FEx rats (Fig. 6b).

Importantly, U46619, a TxA_2_ agonist given at a maximal concentration of 10^−7^ M, contracted these vessels more effectively at 50 mmHg in FEx animals than in FSe animals (Fig. 5c), and the maximum contraction was significantly higher at 150 mmHg in trained female rats than in control female and trained male rats (Fig. 5d).

#### Biomechanical parameters of intramural coronary resistance arteries from the physiologically hypertrophic left ventricle

**Fig. 7:**
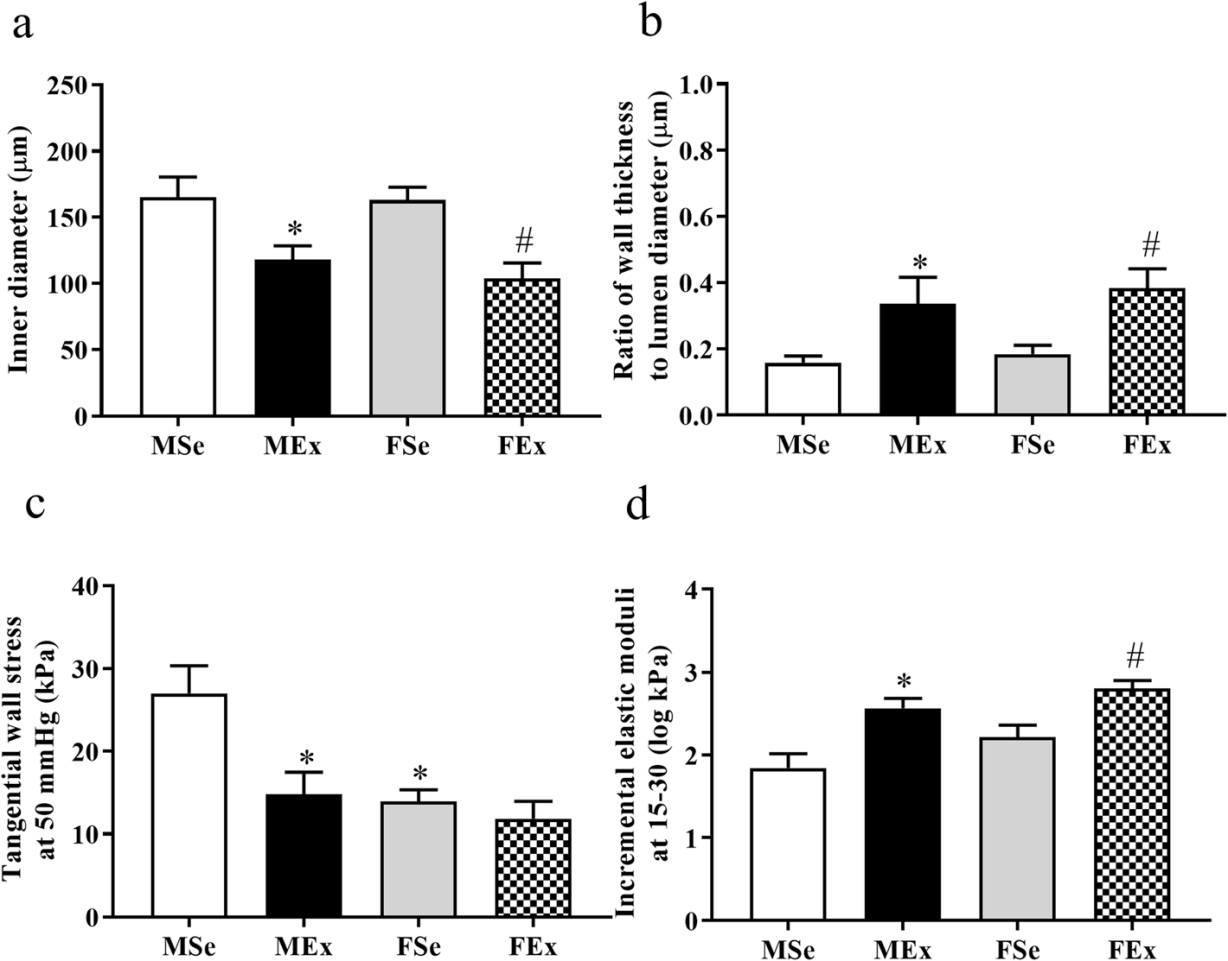
Geometric and biomechanical alterations of coronary resistance artery segments induced by long-term physical exercise. Values were measured in the fully relaxed state. **a** Significantly reduced inner diameters in the male and female trained groups. (*F*_training_ = 19.515, *F*_sex_ = 0.448, *F*_int_ = 0.258, df_training_ = 1, df_sex_ = 1, df_int_ = 1, *P*_training_ < 0.001, *P*_sex_ = 0.509, and *P*_int_ = 0.615). **b** The wall thickness to diameter ratio was significantly elevated after training in both the male and female groups. (*F*_training_ = 14.962, *F*_sex_ = 0.672, *F*_int_ = 0.0526, df_training_ = 1, df_sex_ = 1, df_int_ = 1, *P*_training_ < 0.001, *P*_sex_ = 0.419, and *P*_int_ = 0.842). **c** The tangential wall stress at 50 mmHg of pressure was significantly reduced in trained male animals (close to the level in female animals). FSe rats had lower values compared with MSe rats. (*F*_training_ = 7.499, *F*_sex_ = 9.494, *F*_int_ = 3.382, df_training_ = 1, df_sex_ = 1, df_int_ = 1, *P*_training_ = 0.01, *P*_sex_ = 0.004, and *P*_int_ = 0.076). **d** The incremental elastic modulus at 15–30 kPa of wall stress increased in trained female and trained male animals compared with similar control groups. (*F*_training_ = 25.326, *F*_sex_ = 5.506, *F*_int_ = 0.267, df_training_ = 1, df_sex_ = 1, df_int_ = 1, *P*_training_ < 0.001, *P*_sex_ = 0.023, and *P*_int_ = 0.607). Two-way ANOVA with post hoc Tukey’s test. Values are the means ± SEM. *P < 0.05 vs. MSe; ^#^*P* < 0.05 vs. FSe

There was no significant difference between the relaxed outer diameters (203 ± 13, 204 ± 13, 208 ± 13, and 191 ± 11 μm at 50 mmHg for the MSe, MEx, FSe, and FEx groups, respectively, n.s.), which was the result of careful selection of the specimens from the network. However, in intensively trained male and female rats, the inner diameters of the vessels were reduced in the relaxed state (Fig. 7a), which resulted in an elevated ratio of wall thickness to lumen diameter in both MEx and FEx animals (Fig. 7b).

The tangential wall stress was significantly lower in FSe rats than in MSe rats at physiological pressures (at 50 mmHg). This value significantly decreased in the MEx group approaching the level of that in the female animals (Fig. 7c). There was no significant difference between the tangential wall stresses of the groups at high pressures (45.6 ± 6, 33.7 ± 6, 29.2 ± 3, and 29.6 ± 5 kPa at 100 mmHg, as well as 75.1 ± 11, 52.5 ± 10, 46.7 ± 6, and 42.2 ± 9 kPa at 150 mmHg, for the MSe, MEx, FSe, and FEx groups, respectively, n.s.).

The tangential elastic moduli increased in the long-term swimming-trained male and female animals compared to that of the sedentary animals at 15 and 30 kPa of wall tension (Fig. 7d).

## Discussion

In the present study, we found that the intramural coronary resistance arteries of the rat were structurally and functionally transformed as a result of a long-term intensive exercise training program. While the inner diameter of these arterioles decreased, the ratio of the wall thickness to lumen diameter increased, and the incremental elastic moduli increased in trained animals of both sexes. In addition, tangential wall stress was lower in female animals than in male sedentary animals, and after exercise, tangential wall stress was reduced in male exercised animals to a level approaching that in female animals. Here, we show that there is training-induced remodeling of coronary resistance artery function in both sexes, but in several aspects, this remodeling is different in both sexes. While U46619-induced contractility increased in trained female rats, endothelial-dependent dilatation increased in exercise-trained male rats. The lamina elastica remodeling was different in female and male animals in the exercise group. To our knowledge, no publication about the sex differences in exercise-induced remodeling of resistance-sized coronary arteries has been published previously.

### Similarities in exercise-induced heart and coronary adaptation in male and female rats

In agreement with previous results of the rodent model of exercise-induced cardiac hypertrophy, an increased heart weight/body weight was observed in response to the swimming-training program in both male and female rats [7, 24]. Our 12-week swim training program can be considered intensive, as shown by the degree of ventricular hypertrophy developed in both males and females. This ventricular hypertrophy was demonstrated by marked elevations in the heart weight/body weight ratio, which were associated with systolic functional improvement indicated by an increased ejection fraction and fractional shortening. No sex differences could be identified regarding systolic ventricular function in our study. These alterations are characteristic of adaptation to long-term exercise and might be a consequence of the hypertrophy of ventricular myocytes [1, 3, 25].

Remodeling of the vessels by physical exercise develops, at least in part, as an effect of elevated endothelial shear stress, transiently elevated pressures in the lumen, circumferential wall stress, and metabolic signals [11, 12, 26]. In our in vitro studies, we did not find a difference in the spontaneous tone of segments from the different groups at 50 mmHg intraluminal pressure.

Another important aspect of vascular remodeling is the alteration of wall geometry. During the preparation process, we selected 2–3-mm-long segments from the subsurface branches of the left anterior descending coronary resistance artery that had approximately 200-μm outer diameters. Our attempt was successful, as demonstrated by the fact that the outer diameters were almost identical in all four groups. The inner diameter decreased, and the wall thickness to diameter ratio increased in trained animals. Several observations have described lumen distension and thinner walls in vessels as effects of long-term physical exercise [11, 12, 26–29], while others, similarly to us, have found thicker walls and decreased lumens [14, 30, 31]. A possible explanation is that different segments of the resistance artery network are affected in different manners during long-term exercise-induced microcirculatory network remodeling. The elevated wall thickness to lumen diameter ratio may be advantageous in providing better control of the vascular lumen and segmental hemodynamic resistance. That might be needed to ensure a higher level of ‘vasomotion,’ that is, a greater difference between the maximally contracted and relaxed states. The lower values of isobaric wall stress might be advantageous for reducing wall tissue damage when more proximal arteries dilate during physical work and allow the high pulsatile pressure to reach more distal parts of the network. As noted above, the coronary arteries of female rats have been shown to be more effective in this respect than those of male rats.

Geometric remodeling with an elevated wall thickness to lumen diameter ratio was accompanied by remodeling of the elastic properties. At high wall tangential stress values (15–30 kPa), the tangential modulus increased in both sexes. Remodeling of the inner elastic membrane might be responsible for this alteration, but the inner elastic lamina adaptation was different between the two sexes (discussed below).

### Sex differences in the exercise-induced adaptation of the heart and coronaries between male and female rats

Although female rats had lower body weight and absolute cardiac mass than males (valid both for the exercised and sedentary states), an increase in post-mortem-assessed normalized heart weight to body weight and an increased absolute heart mass were observed after a 12-week-long swimming-training protocol in female rats. This sex difference in body weight and heart weight was comparable with other small animal models of exercise-induced cardiac hypertrophy and human athletes as well [18, 24, 32]. Furthermore, trained female animals also had more pronounced ventricular hypertrophy than male animals, similar to a previous publication [18]. This outcome may be attributed to altered ventricular expression of estrogen receptor β and its stimulation [24] and to different energy substrate availabilities of the two sexes [17]. Similar to our results, Oláh et al. found more pronounced exercise-induced LV hypertrophy in female rats than in male rats; activation of the Akt and myosin heavy chain α (MHC)/β-MCH ratio was greater in swimming female rats than in swimming males, and there were sex differences regarding ERK1/2, S6 and mTOR activation [33].

At 150 mmHg, trained female animals had higher tone in Krebs buffer than their trained male counterparts. A similar situation was observed at maximum contraction, with U46619 in the bath, also at 150 mmHg. We observed that trained female animals had an improved contracting ability of their coronary arterioles at high pressure (150 mmHg) compared to trained male animals. There are conflicting data in the literature regarding how the contractility of coronaries is affected by long-term exercise; specifically, some studies have found increased sensitivity to vasoconstrictor agonists [34], while others have found unaltered [35] and reduced [36, 37] agonist-induced contractions for different coronary specimens. Elevated myogenic tone in trained animals has been described previously [12, 14, 38].

The elevation of endothelium-dependent vasorelaxation by long-term exercise has been described in some earlier publications both in male and female animals [12, 14, 39, 40]. During exercise training, wall shear stress is elevated and acts on the endothelial cell layer, inducing acute and chronic adaptation mechanisms, such as enhanced endothelial NO release or decreased endothelin levels in vascular smooth muscle cells [14]. Furthermore, exercise training-induced enhanced endothelium-dependent vasorelaxation is partly due to increased expression of SOD-1 and eNOS [40, 41]. In addition, coronary endothelial cells—rather than cardiomyocytes—play a key role in the enhanced eNOS-dependent relaxation induced by long-term exercise training [42]. Bradykinin-induced relaxation was greater in trained male rats only, indicating an improved endothelial-dependent dilatation effect of exercise. Sex differences in the endothelium-dependent relaxation ability induced by exercise training in the literature are not unknown; these and other publications show that endothelial dilation might be different in specimens of different sexes. There are, however, still several contradictions. Physical exercise increased endothelial relaxation in human peripheral arteries [43, 44]. It enhanced nonendothelial (adenosine-induced) coronary vasodilator capacity [45] but did not seem to affect endothelium-dependent vasorelaxation [29, 46]. Using a much more reduced intensity exercise program than ours, Szekeres et al. also described improved endothelium-dependent vasorelaxation in the low pressure range in intramural coronary resistance arteries of male rats [14]. Similarly, others have found that exercise improved endothelial function in the brachial artery of men, while studies on humans did not confirm the same effect in women [47, 48]. Furthermore, In-Chang Hwang et al. investigated the acute exercise effect and sex differences in flow-mediated dilatation and found that it was reduced in women but not in men in the brachial artery [49].

The tangential wall stress decreased more in the trained male group, approaching the level found in the female animals. Without exercise, significant sex differences were found between the sedentary male and female controls at 50 mmHg, and tangential wall stress was significantly lower in the FSe rats than in the MSe rats. The optical density of noncontractile fibers on resorcin-fuchsin-stained sections was significantly lower in female animals (FSe and FEx) than in MSe rats, and this value was significantly reduced in the MEx group approaching the level of that in the female animals. The higher levels of noncontractile fiber elements observed in male control animals may be connected with the higher tangential wall stress. The lower fiber density in female animals may be connected with the lower tangential wall stress. The swim training in male rats results in a decrease in these fibers, which is again connected with a lower tangential wall stress. The amount of collagen and smooth muscle did not differ between the groups.

The alteration of the inner elastic membrane might be responsible for the elevated tangential modulus in trained male and female animals. In the FEx animals, the distance of the layer with maximal intensity was located farther from the endothelial surface. The inner elastic membrane became thicker in the MEx animals than that in the MSe and FEx rats. Thickening of the elastic membranes as an effect of long-term exercise in the aorta has been described previously by Souza et al. in male rats [31]. Following a moderate training program, Hanna et al. described a reduction in the ‘indentation’ elastic moduli of coronary arterioles, with no change in the collagen/elastic tissue ratio. In a recent study, Szekeres et al. found decreased elastic moduli after moderate exercise in male rats but only in the low pressure range [14, 38]. We are convinced that our pressure arteriography technique offers a better way to show the in vivo tangential elasticity than the indentation modulus, which measures elasticity in the radial direction. We must admit that because of the substantial myogenic tone of the segments, the elasticity measurements could be performed only in the relaxed state.

The substantial sex differences in long-term exercise adaptation that we found might be due to the direct effects of sex hormones on the vascular walls. Estrogen receptor β seems to be responsible for at least part of the sex differences in protein expression in the heart that are found in connection with intensive training [18, 24]. Another explanation could be that there is different energy-substrate availability [17]. Sex affects the transmembrane flow of Ca^2+^ in coronary vascular smooth muscle cells [50]. Sex differences between males and females have been revealed in the activity of protein kinase C enzyme in the coronary artery walls after intensive training [51]. This question has great clinical and epidemiological significance, as differences in the control of vascular wall remodeling might explain the well-known lower level of cardiovascular morbidity in females compared to males, which is effective until menopause [52, 53]. Testosterone, both at high and low doses alike, has a risk-elevating effect on vascular function. At high doses, it impairs endothelial function and increases the likelihood of acute coronary events. At too low doses, it increases the risk of stroke and coronary heart disease. At optimal levels, however, testosterone increases the vasodilator response to NO on the coronary artery wall [52, 54, 55].

The morphological and biomechanical adaptation of coronary arteries may serve as a response to more intensive vascular reactivity changes that are induced by regular exercise. Initially, there were contradictory observations in the literature on the responsiveness induced by different pharmacons, but it later became apparent that the vasomotor regulation of large epicardial coronaries is independent of intramural resistance arterioles [29]. Thus, the contradictory results may have been due to the different sizes or types of vascular segments and to the different vascular beds. Currently, we know that not only the vasomotor regulation of large and small coronary arteries but also the exercise-induced adaptation of these arteries is different. Human and animal studies have shown that the resting basal tone and endothelial dilation capacity of the coronary arteries of trained individuals is greater than that of the coronary arteries of sedentary individuals both in males and females [14, 56, 57]. During exercise, increased pulsatile pressure increases shear stress, which activates the endothelial surface of blood vessels, inducing acute and chronic adaptation mechanisms, such as increased endothelial NO release or decreased endothelin secretion in smooth muscle cells in male rats [14]. A further explanation for the increased endothelial dilation observed in athletic animals is the increased expression of SOD-1 and eNOS that results from long-term exercise in male and female swine [40, 41].

A limitation of our study is that it was performed on relatively young animals, and the conclusions might not apply directly to coronary remodeling in older mammals with slower adaptation processes. In elderly individuals, due to anabolic resistance, the body is less adaptive, and many diseases affect the ability to perform regular physical activity.

### Perspectives and significance

Our observations prove that the resistance coronary arteries are adapted structurally and functionally to long-term intensive swimming exercise, as ventricular myocardium forms the ‘athlete’s heart.’ Furthermore, our study proves that these sport adaptations have similarities and differences in both sexes.

Regular participation in sports has a very positive effect on the treatment of various cardiovascular diseases, and it would be worthwhile to study exercise-induced coronary adaptation in different diseases. The current study should be continued by direct observations of sex hormones on the cardiovascular effects of training. In addition, our studies were restricted to a single segment of the coronary resistance artery network, while local vascular resistance is determined by the properties of the whole resistance artery network. Network studies should extend the picture of the sex effects of exercise training to the different segments of the coronary artery tree.

## Conclusions

Our observations prove that if subjected to an intense chronic training program, not only the heart but also the coronary vessels will be affected, and these alterations have similarities and differences in the male and female sexes. The remodeled coronary resistance artery wall presented with lower tangential wall stress both in trained females and males. The coronary resistance arteries walls’ elastic modulus at physiological pressures increased in both sexes but with different histological remodeling: in males, the density of the inner elastic membrane increased, and in females, it was dislocated toward the adventitia. Spontaneous and agonist-induced arterial tone was more developed in trained females at higher pressures and less developed in males. Endothelial dilatation increased more in trained male rats than in trained female rats. Such differences should be taken into consideration when evaluating the effects of long-term exercise on the functional performance and prevention of pathologies in coronary resistance arteries. Our conclusion is that the biomechanics of coronary arterioles adapted to long-term exercise. The observed similarities and sex differences in the coronary resistance artery biomechanics of rats with physiological LV hypertrophy may contribute to a better understanding of physiological and pathological coronary function in active and retired athletes of both sexes.

## Data Availability

All data generated or analyzed during this study are included in this published article [and its supplementary information files].
